# Giant appendiceal mucocele (17 cm) treated by laparoscopic appendectomy: a rare presentation and review of the literature

**DOI:** 10.1093/jscr/rjaf864

**Published:** 2025-10-31

**Authors:** Kanan Ismayilzada, Koray Topgul

**Affiliations:** Department of General Surgery, Medicana Ataşehir International Hospital, Ataşehir, Istanbul 34758, Turkey; Department of General Surgery, Medicana Ataşehir International Hospital, Ataşehir, Istanbul 34758, Turkey

**Keywords:** appendiceal mucocele, giant appendix, laparoscopic appendectomy, LAMN, case report

## Abstract

Appendiceal mucocele is an uncommon clinical entity, usually detected incidentally and rarely exceeding 10 cm in length. We present a unique case of a giant 17-cm appendiceal mucocele successfully managed with laparoscopic appendectomy. A 51-year-old woman presented with abdominal bloating, and colonoscopy revealed a large submucosal lesion at the cecal base. Computed tomography demonstrated a 17-cm cystic lesion consistent with appendiceal mucocele. The patient underwent laparoscopic appendectomy without complications, and histopathological analysis confirmed low-grade appendiceal mucinous neoplasm. She was discharged uneventfully on the first postoperative day and remained asymptomatic at follow-up. Giant mucoceles are exceptionally rare and carry a significant risk of rupture and pseudomyxoma peritonei, emphasizing the importance of careful surgical management. This case demonstrates that even extraordinarily large mucoceles can be safely and effectively treated laparoscopically, supporting the role of minimally invasive surgery as a feasible and reliable option in such high-risk cases.

## Introduction

Appendiceal mucocele is an uncommon pathological entity characterized by abnormal dilatation of the appendix caused by intraluminal mucin accumulation. It accounts for less than 0.3% of all appendectomy specimens and represents a wide pathological spectrum ranging from simple retention cysts to low-grade appendiceal mucinous neoplasms (LAMN) and adenocarcinoma [1, 2].

Although often discovered incidentally, appendiceal mucoceles are clinically important due to their potential complications. Rupture may result in pseudomyxoma peritonei, a condition associated with significant morbidity and poor prognosis if not appropriately treated [3, 4]. Preoperative diagnosis is difficult because symptoms are vague and imaging may mimic cecal or adnexal lesions [4].

Surgical resection remains the definitive treatment, aiming to prevent rupture and achieve complete removal. While open surgery has traditionally been considered the standard approach, the role of laparoscopy in managing large mucoceles has been debated because of concerns regarding intraoperative spillage. However, recent evidence suggests that laparoscopic appendectomy can be performed safely with careful surgical technique [5, 6].

Herein, we present a rare case of a giant 17-cm appendiceal mucocele treated successfully with laparoscopic appendectomy. To our knowledge, few cases of this size have been reported [6, 7]. This case highlights both the rarity of such exceptionally large lesions and the feasibility of minimally invasive surgery in their management.

## Case presentation

**Figure 1:**
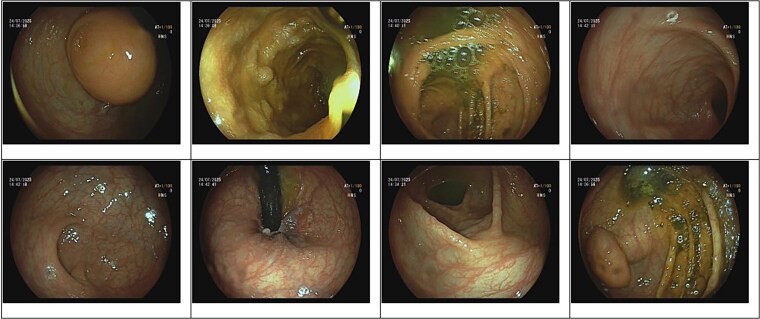
Colonoscopy image demonstrating a submucosal lesion at the cecal base, suggestive of an appendiceal origin.

A 51-year-old woman with no prior medical or surgical history presented with a 1-month history of indigestion and abdominal bloating. Physical examination and laboratory findings were unremarkable. Colonoscopy demonstrated a submucosal lesion, approximately 7–8 cm in size, at the cecal base near the appendiceal orifice (Fig. 1). As the lesion could not be fully characterized, abdominal computed tomography (CT) was performed and revealed a sharply marginated, mildly lobulated cystic lesion in the appendiceal region, measuring 17 cm in length and up to 3.5 cm in maximal distal diameter, containing dense fluid material. The appearance was most consistent with an appendiceal mucocele or mucinous cystadenoma. Although CT confirmed the diagnosis, the imaging files were not available for inclusion in this report.

**Figure 2:**
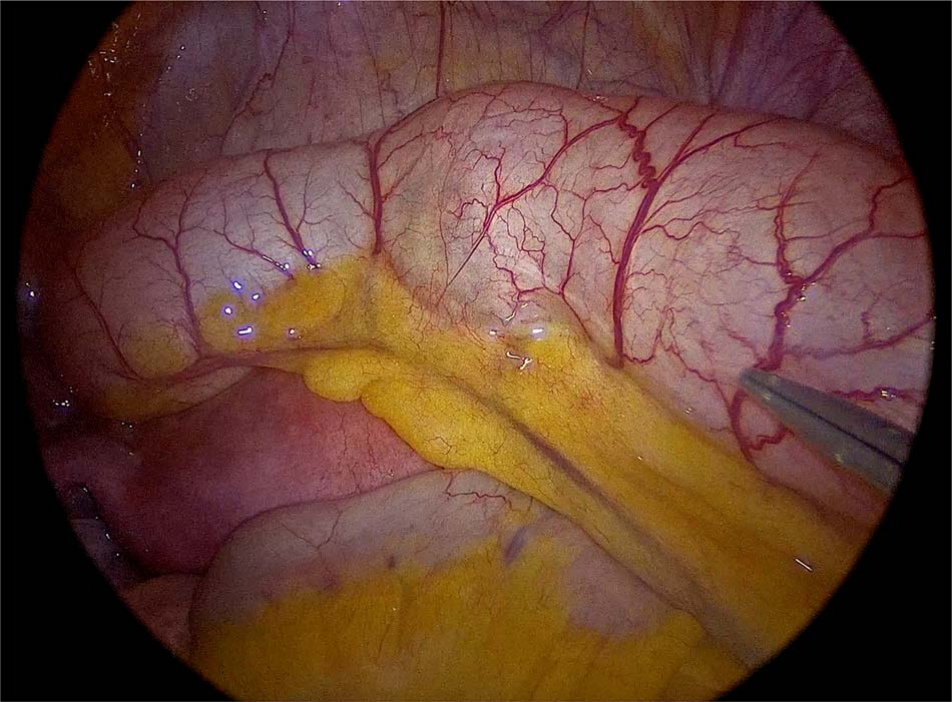
Laparoscopic view demonstrating the markedly distended appendix consistent with a giant mucocele.

**Figure 3:**
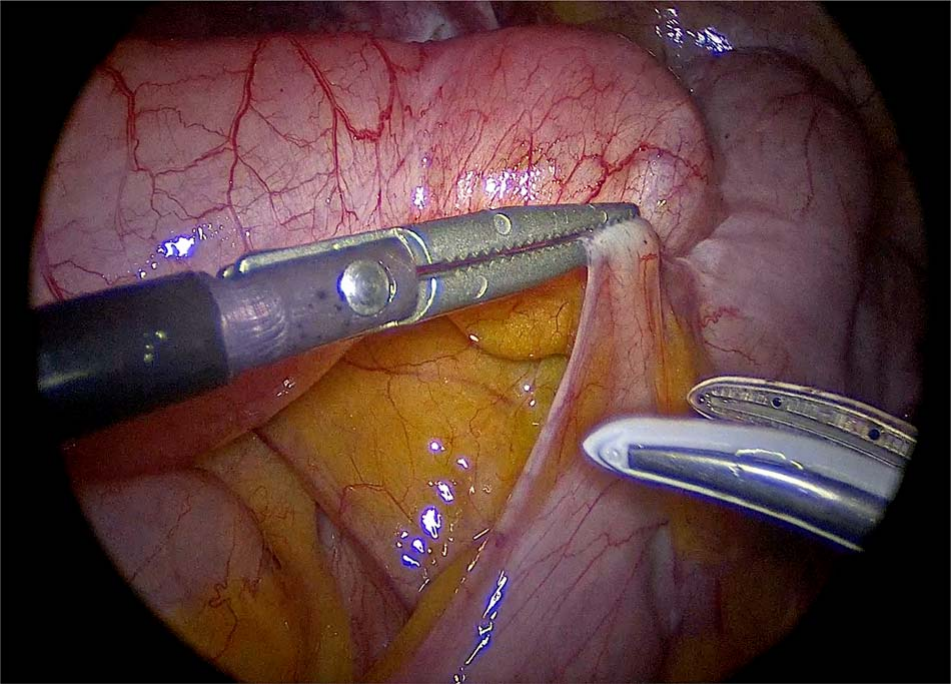
Careful laparoscopic dissection of the appendiceal base, with meticulous attention to avoid rupture of the specimen.

**Figure 4:**
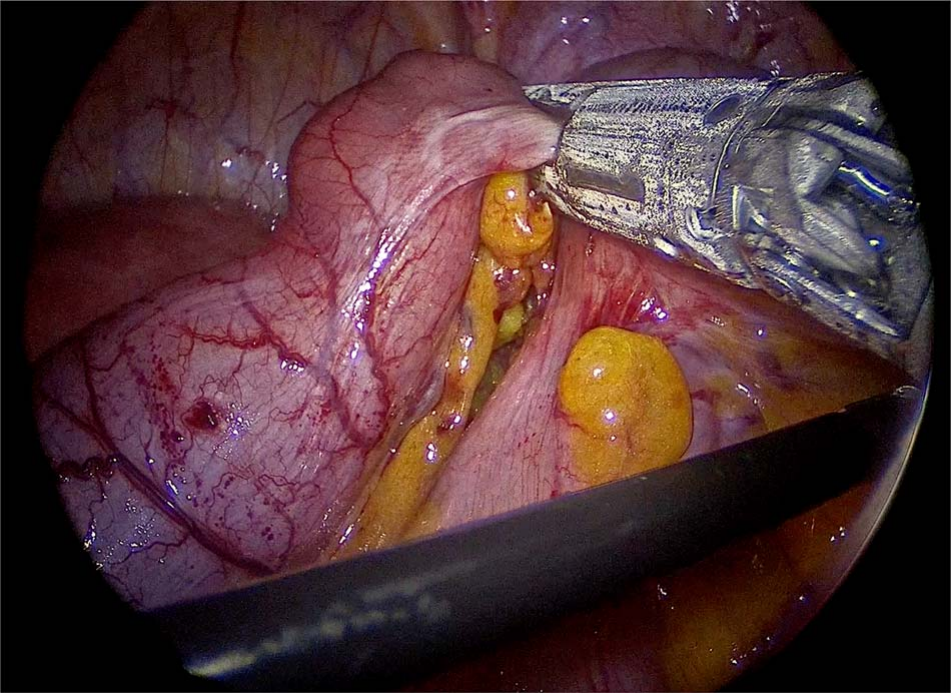
Division of the appendiceal base with an Endo-GIA stapler, including the cecal base while preserving the ileocecal valve.

**Figure 5:**
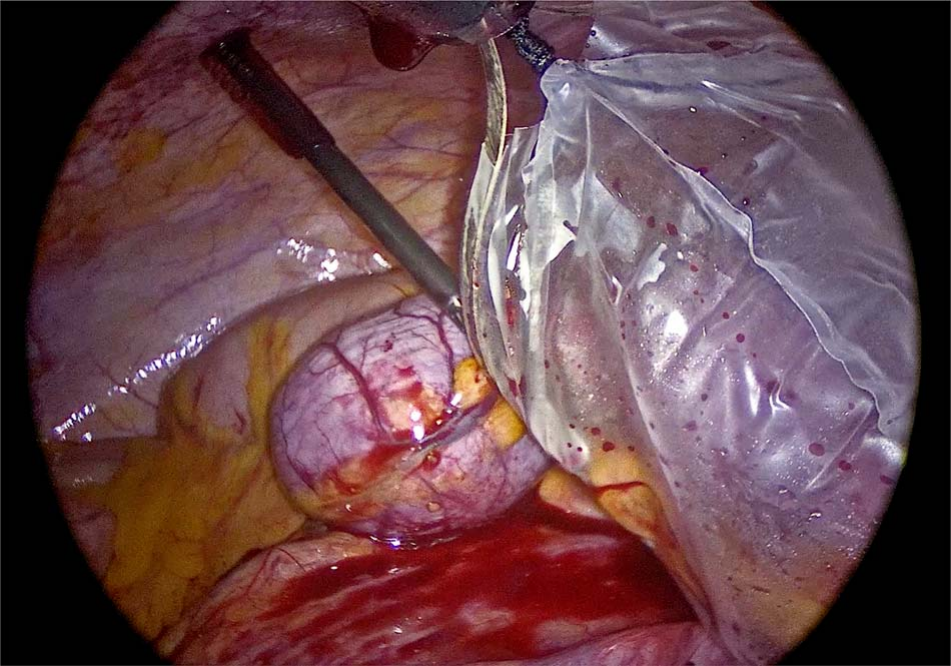
Intact retrieval of the specimen within an endoscopic bag through the umbilical incision to prevent rupture or intraperitoneal spillage.

**Figure 6:**
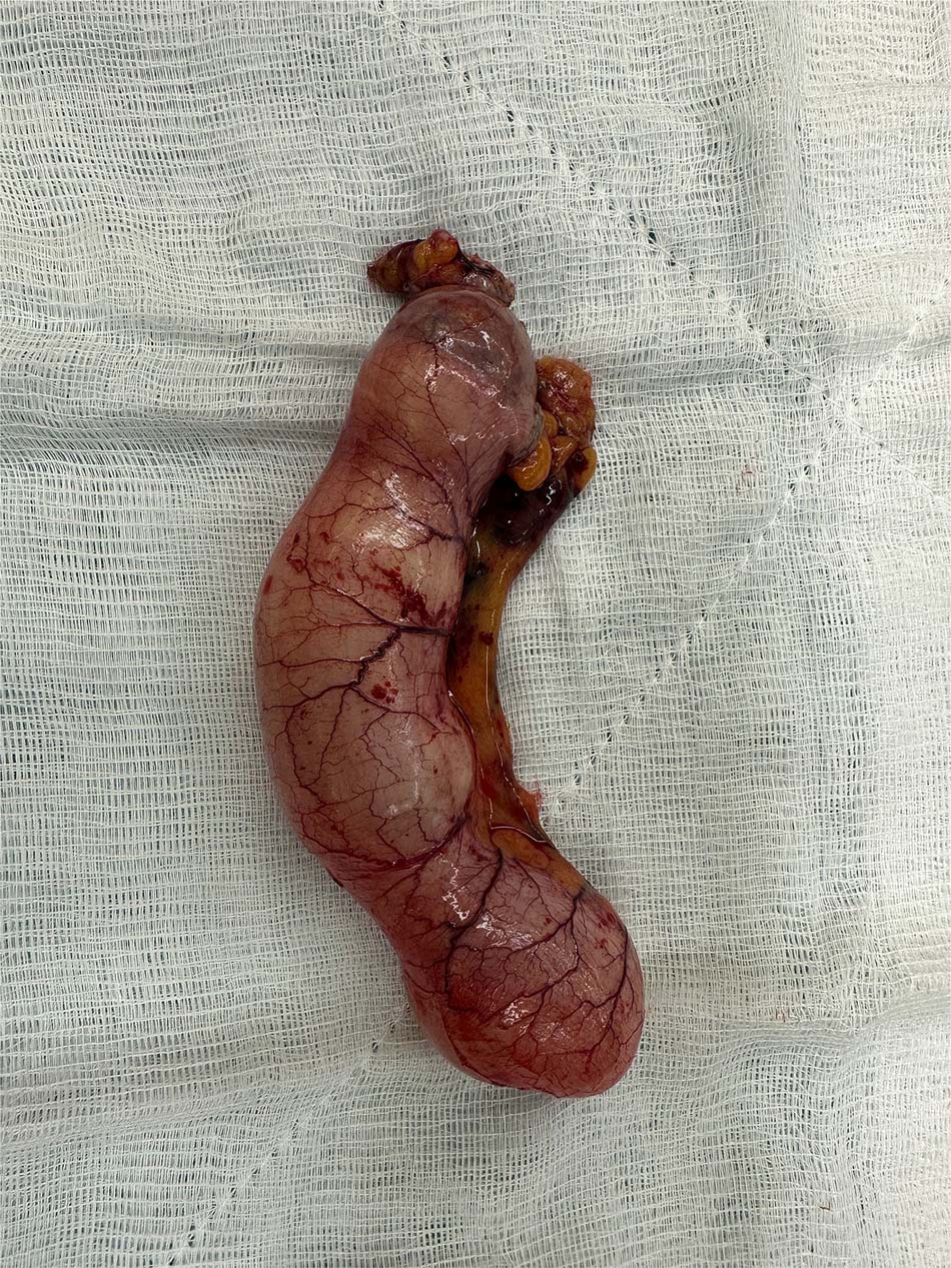
Gross specimen of the resected appendix measuring approximately 17 cm in length, consistent with a giant appendiceal mucocele.

Laparoscopic exploration was undertaken using three ports: a 10-mm umbilical trocar, a 5-mm suprapubic trocar, and a 12-mm trocar in the right upper quadrant along the Spigelian line. A markedly distended appendix consistent with a giant mucocele was identified (Fig. 2). Meticulous dissection was performed with great care to avoid rupture of the specimen (Fig. 3). Appendectomy was then completed with an Endo-GIA stapler, ensuring inclusion of the cecal base while preserving the ileocecal valve (Fig. 4). The specimen was retrieved intact within an endoscopic bag through a slightly extended umbilical incision to prevent spillage (Fig. 5). Gross examination confirmed a 17-cm cystically dilated appendix, consistent with a giant mucocele (Fig. 6).

The postoperative course was uneventful, and the patient was discharged on the first postoperative day. Histopathological examination confirmed LAMN. At 10-day and 1-month follow-up visits, the patient was asymptomatic and reported resolution of her symptoms.

## Discussion

Appendiceal mucoceles are rare, accounting for fewer than 0.3% of appendectomy specimens [1, 7]. Most are <10 cm, whereas lesions >15 cm are extremely uncommon [6, 8]. Our 17-cm case is among the largest reported.

The main clinical concern is rupture, which may lead to pseudomyxoma peritonei and significant morbidity [9, 10]. This risk underscores the need for early recognition and careful management. Diagnosis can be difficult, as patients often present with vague symptoms and imaging may mimic adnexal or cecal tumors [11].

Open surgery has traditionally been recommended to minimize rupture risk [12]. Laparoscopy, once controversial, is now accepted as safe when meticulous technique is applied. Critical precautions include minimal handling, stapler use for secure resection at the cecal base, and intact retrieval with an endoscopic bag [5].

Several cases illustrate this trend: Rampone *et al.* [6] reported a 15-cm mucocele treated by open resection, Nogueira *et al.* [5] described laparoscopic management of a giant cystadenoma, and Ma *et al.* [7] more recently presented a 16-cm LAMN excised laparoscopically without complications. Our case adds to this evidence, demonstrating that even a 17-cm mucocele can be safely managed laparoscopically when strict precautions are observed.

Thus, careful patient selection and meticulous technique are key. Laparoscopy, once avoided in giant mucoceles, may represent a safe and effective option in appropriately selected cases.

## Conclusion

Giant appendiceal mucoceles are exceptionally rare and carry a substantial risk of rupture and pseudomyxoma peritonei. Our case demonstrates that even a 17-cm lesion—one of the largest reported to date—can be managed successfully with a laparoscopic approach. This highlights the feasibility and safety of minimally invasive surgery when meticulous surgical technique and appropriate precautions are applied.
